# Targeting the SPOCK1-snail/slug axis-mediated epithelial-to-mesenchymal transition by apigenin contributes to repression of prostate cancer metastasis

**DOI:** 10.1186/s13046-019-1247-3

**Published:** 2019-06-10

**Authors:** Ming-Hsien Chien, Yung-Wei Lin, Yu-Ching Wen, Yi-Chieh Yang, Michael Hsiao, Junn-Liang Chang, Hsiang-Ching Huang, Wei-Jiunn Lee

**Affiliations:** 1 Graduate Institute of Clinical Medicine, College of Medicine, Taipei Medical University, Taipei, Taiwan; 2 TMU Research Center of Cancer Translational Medicine, Taipei Medical University, Taipei, Taiwan; 3 Pulmonary Research Center, Wan Fang Hospital, |Taipei Medical University, Taipei, Taiwan; 4 Department of Urology, School of Medicine, College of Medicine, Taipei Medical University, 250 Wu-Hsing Street, Taipei, 11031 Taiwan; 5 Department of Urology, Wan Fang Hospital, Taipei Medical University, Taipei, Taiwan; 6 The Genomics Research Center, Academia Sinica, Taipei, Taiwan; 7 Department of Pathology and Laboratory Medicine, Taoyuan Armed Forces General Hospital, Taoyuan, Taiwan; 8 Biomedical Engineering Department, Ming Chuan University, Taoyuan, Taiwan; 9 Graduate Institute of Medical Sciences, College of Medicine, Taipei Medical University, Taipei, Taiwan; 10 Department of Medical Education and Research, Wan Fang Hospital, Taipei Medical University, Taipei, Taiwan; 11 Cancer Center, Wan Fang Hospital, Taipei Medical University, Taipei, Taiwan

**Keywords:** Prostate cancer, SPOCK1, Metastasis, Snail, Slug, Epithelial-to-mesenchymal transition, Apigenin

## Abstract

**Background:**

Prostate cancer (PCa) is considered one of the most prevalent malignancy globally, and metastasis is a major cause of death. Apigenin (API) is a dietary flavonoid which exerts an antimetastatic effect in various cancer types. Sparc/osteonectin, cwcv, and kazal-like domains proteoglycan 1 (SPOCK1) is a crucial modulator of tumor growth and metastasis in cancers. However, the role and underlying regulatory mechanisms of SPOCK1 in the API-mediated antimetastatic effects of PCa remain unclear.

**Methods:**

MTS, colony formation, wound-healing, and transwell assays were conducted to evaluate the effects of API on PCa cell proliferative, migratory, and invasive potentials. In vivo orthotopic bioluminescent xenograft model were employed to determine antitumor activity of API. PCa cells were transfected with either Snail-, Slug-, SPOCK1-overexpressing vector, or small hairpin (sh)SPOCK1 to determine the invasive abilities and expression levels of SPOCK1 and epithelial-to-mesenchymal transition (EMT) biomarkers in response to API treatment. Immunohistochemical (IHC) assays were carried out to evaluate the expression level of SPOCK1 in PCa xenografts and a PCa tissue array. Associations of SPOCK1 expression with clinicopathological features and prognoses of patients with PCa were analyzed by GEO or TCGA RNA-sequencing data.

**Results:**

API significantly suppressed in vitro PCa cell proliferation, migration, and invasion and inhibited in vivo PCa tumor growth and metastasis. Moreover, survival times of animals were also prolonged after API treatment. Mechanistic studies revealed that API treatment resulted in downregulation of SPOCK1, which was accompanied by reduced expressions of mesenchymal markers and subsequent attenuation of invasive abilities of PCa cells. Overexpression of SPOCK1 in PCa xenografts resulted in significant promotion of tumor progression and relieved the anticancer activities induced by API, whereas knockdown of SPOCK1 had opposite effects. In clinical, SPOCK1 levels were higher in tumor tissues compared to non-tumor tissues, which was also significantly correlated with shorter disease-free survival in PCa patients.

**Conclusions:**

Levels of SPOCK1 increase with the progression of human PCa which suggests that SPOCK1 may act as a prognostic marker or therapeutic target for patients with PCa. Suppression of SPOCK1-mediated EMT signaling contributes to the antiproliferative and antimetastatic activities of API in vitro and in vivo.

**Electronic supplementary material:**

The online version of this article (10.1186/s13046-019-1247-3) contains supplementary material, which is available to authorized users.

## Background

Prostate cancer (PCa) is the most commonly diagnosed cancer in males and the second leading cause of cancer-associated deaths worldwide [1]. Androgen deprivation therapy (ADT) remains the primary clinical treatment for patients in the early stage of PCa. However, despite androgen ablation, nearly all patients with advanced stages of PCa experience recurrence as the disease develops into castration-resistant PCa (CRPC), which is characterized by aggressive growth and distal organ metastasis, and is incurable [2]. Therefore, early diagnosis and treatment before the tumor metastasizes are critical for improving survival of patients with PCa. Despite advances in detection and treatment strategies, there are currently no effective therapeutics to treat metastatic PCa. Hence, the means to prevent PCa progression and conduct necessary interventions before the cancer has spread to other organs remain major clinical challenges.

The epithelial-to-mesenchymal transition (EMT) is a biological process, and activation of the EMT program contributes to cell invasion and metastasis in multiple cancers [3]. Clinical evidence suggests that aberrant activation of the EMT is correlated with therapeutic resistance and tumor aggressiveness and negatively impacts survival of patients with PCa [4]. One oncogene, *Sparc/osteonectin, cwcv, and kazal-like domains proteoglycan 1 (SPOCK1)*, was observed to affect the EMT process by facilitating metastasis in various cancers [5, 6]. SPOCK1, also known as testican-1, is a proteoglycan that belongs to a Ca^2+^-binding proteoglycan family that was implicated in cell proliferation, DNA replication, apoptosis, and the migration and invasion of cancer cells [7]. More importantly, clinicopathologic analyses revealed that SPOCK1 is frequently overexpressed in PCa tissues [8], and is involved in cancer recurrence, drug resistance, and metastasis [8, 9]. However, in light of our current knowledge, the function of SPOCK1 in PCa metastasis is not entirely understood, and even less is known about the underlying mechanism responsible for the SPOCK1-mediated EMT process in PCa development and progression.

Natural products and their derivatives have been further developed as anticancer agents and provide potential sources for novel drugs to treat cancer [10]. Flavonoids are naturally occurring polyphenolic metabolites, which are present in a wide variety of edible plant sources, such as fruits, vegetables, grains, nuts, seeds, tea, and traditional medicinal herbs [11]. Epidemiological studies and systematic analyses have shown an inverse association between the dietary intake of flavonoids and the risk of cancer [12]. Apigenin (API), 4′,5,7-trihydroxyflavone, is one of the most common flavonoids and is found in significant quantities in a variety of vegetables and fruits. Accumulating evidence has revealed that the anticancer properties of API are due to its ability to cause cell cycle arrest, trigger apoptosis, induce autophagy, inhibit migration/invasion, attenuate drug resistance, and stimulate immune responses in various cancer types in vitro and in vivo [13, 14]. API was recently shown to effectively suppress migration and invasion through modulation of the EMT process in PCa [15]. Recently, we also showed that API suppresses CD26/DPPIV expression, and the interplay between p-Akt and Snail/Slug contributed to inhibition of the EMT-mediated invasive ability and subsequently blocked tumor metastasis in a human A549 xenograft model [16].

Although API was shown to be a promising molecule for use in PCa prevention and therapy [17], the precise mechanism underlying its modulation of cell motility and the antimetastatic effects of API so far remain underexplored. In the present study, we found that SPOCK1, which is upregulated in PCa, is involved in the invasion and metastasis of cancer cells and was correlated with poor prognoses. More importantly, we demonstrate for the first time that API suppressed SPOCK1 expression leading to attenuation of PCa metastasis by targeting the Snail/Slug-mediated EMT process.

## Methods

### Materials

API (A3145) and dimethyl sulfoxide (DMSO) were purchased from Sigma-Aldrich (St. Louis, MO). Fetal bovine serum (FBS), antibiotics, molecular weight standards, trypsin-EDTA, and all medium additives were obtained from Life Technologies (Gaithersburg, MD). The CellTiter 96 AQueous One Solution Proliferation Assay System was purchased from Promega (Madison, WI). An enhanced chemiluminescence (ECL) kit was purchased from Amersham (Arlington Heights, IL). Antibodies specific for fibronectin (ab2413) and SPOCK1 (ab229935) were obtained from Abcam (Cambridge, MA). Antibodies specific for cleaved-PARP (#9541), phospho-Ak (ser473, #9271), Akt (#4691), Snail (#3895), Slug (#9585), Twist (#46702), and E-cadherin (#3195) were obtained from Cell Signaling Technology (Danvers, MA). Antibodies specific for vimentin (550513) and N-cadherin (610920) were purchased from BD Biosciences (San Jose, CA). Antibodies specific for cyclin D1 (sc-8396), cyclin E (sc-377,100), β-actin (sc-47,778), and goat anti-rabbit (sc-2004) and anti-mouse (sc-2005) immunoglobulin G (IgG) antibodies were obtained from Santa Cruz Biotechnology (Santa Cruz, CA). Polyvinylidene fluoride (PVDF) membranes for Western blotting were purchased from Bio-Rad (Hercules, CA). Unless otherwise specified, other chemicals used in this study were purchased from Sigma Chemical (St. Louis, MO).

### Cell lines and cell culture

The human transformed benign prostate cell line PNT2 and human PCa cell lines including metastatic hormone-sensitive cell line (LNCap) and metastatic CRPC cell lines (DU145, PC-3, and PC-3 M) were all purchased from American Type Culture Collection (ATCC, Manassas, VA). PNT2 cells were cultured in RPMI 1640 medium (Gibco BRL); PC-3 and PC-3 M cells were cultured in minimal essential medium (MEM; Gibco BRL, Grand Island, NY); DU145 cells were cultured in Dulbecco’s modified Eagle’s medium (DMEM; Gibco BRL); and LNCap cells were maintained in T-medium (Gibco BRL) supplemented with 10% FBS (Invitrogen) and 1% penicillin-streptomycin-glutamine (Invitrogen). Cells were regular passaged at 70%~ 80% confluence via trypsinization using 1 × trypsin and 0.05% EDTA followed by resuspension in complete medium. All cells were incubated at 37 °C in a 5% CO_2_ and 95% air atmosphere.

### Cell viability assay

LNCap, DU145, PC-3, and PC-3 M human PCa cells (5 × 10^3^) were seeded onto 96-well plates in 100 μL of culture medium. After 24 h of incubation to allow cells to adhere, cells were treated with various concentrations of API (0~80 μM) for 24, 48, and 72 h and then subjected to a cell-viability assay (MTS assay; Promega, Madison WI). The absorbance was measured at 490 nm using a microplate reader. Values are the mean ± standard deviation (SD) of triplicate wells and were normalized to that of the control group to determine the percent viability. Values of the half maximal inhibitory concentration (IC_50_) were determined through the dose-response curves by using GraphPad Prism 6.0 (GraphPad software, La Jolla, CA).

### Colony formation assay

Human PCa cells were diluted and seeded at 1000 cells/well in 6-well culture plates. After 24 h, cells were treated with various concentrations of API (0~80 μM) or the vehicle for 48 h and then cultured under standard conditions for 10~14 days. The medium was replaced every 3 days. Finally, colonies were stained with 0.1% crystal violet, and colonies with more than 50 cells were counted.

### Wound healing assays

PC-3 M and DU145 cells were grown to full confluence in 6-well plates and a small area was then disrupted by scratching the monolayer with a 200-μl plastic pipette tip. Cells were washed twice with phosphate-buffered saline (PBS) and replaced with complete medium containing various concentrations of API, and wound closure was observed after 48 h. Images were immediately captured under a phase-contrast microscope at 100x magnification, while at 48 h, cells were washed with PBS and then fixed with 4% paraformaldehyde followed by staining with 0.1% crystal violet.

### Transwell migration and invasion assays

Cell motility was analyzed with the aid of a transwell. To analyze cell migration, 3 × 10^4^ cells in 0.2 ml of serum-free medium were seeded in an uncoated top chamber (24-well insert; pore size, 8 μm; Corning Costar, Corning, NY), and medium supplemented with 10% FBS in the lower chamber was used as a chemoattractant. An invasion assay was conducted following the same procedure, with the exception that 5 × 10^4^ cells were plated in a Matrigel (BD Biosciences, Bedford, MA)-coated top chamber. After 48 h of incubation, cells that had migrated or invaded to the bottom surface of the insert were fixed in 100% methanol for 5 min, stained in 0.1% crystal violet for 30 min, and rinsed in PBS, and cells on the top surface of the insert were removed by wiping with a cotton swab. The number of cells migrating or invading through the membrane was visualized and counted under a light microscope (200×, three random fields per well).

### Western blot analysis

Cells were washed with PBS plus zinc ions (1 mM), and lysed with lysis buffer (10 mmol/l Tris-hydrochloride, 0.25 mol/l sucrose, 5 mmol/l EDTA, 50 mmol/l NaCl, 30 mmol/l sodium pyrophosphate, 50 mmol/l NaF, 1 mmol/l Na_3_VO_4_, 1 mmol/l PMSF, and 2% protease inhibitor cocktail; at pH 7.5). The protein concentration in the resulting lysate was determined with a bicinchoninic acid protein assay (Merck, Darmstadt, Germany). Appropriate quantities of protein (30~50 μg) were separated by sodium dodecyl sulphate-polyacrylamide gel electrophoresis (SDS-PAGE) or 4–20% gradient SDS-PAGE (TOOLS, New Taipei City, Taiwan) and transferred onto nitrocellulose membranes. The membranes were inhibited and incubated overnight with the appropriate primary antibody at dilutions according to the manufacturer’s instructions. The membranes were subsequently washed and incubated with the corresponding horseradish peroxidase-conjugated secondary antibody at a 1:1000 dilution in Tris-buffered saline-Tween 20 (10 mM Tris-HCl at pH 7.4, 150 mM NaCl and 0.1% Tween-20). The bound secondary antibody was then detected using an ECL system (Pierce Biotechnology, Rockford, IL).

### Transient transfection of DNA

To overexpress Snail, or Slug [18], semiconfluent cultures of PCa cells in a 6-mm^2^ Petri dish were transfected with 3 μg of an empty or expression vector using the Lipofectamine 3000 Transfection Reagent (Invitrogen, Carlsbad, CA) for 6 h according to the manufacturer’s instructions. At 24 h after transfection, cells were analyzed for invasion/migration and Snail and Slug expressions.

### Lentiviral production and infection

The short hairpin (sh)RNA oligonucleotide sequence specifically targeting human *SPOCK1* gene messenger (m)RNA was purchased from the National RNAi Core Facility at Academic Sinica (Taipei, Taiwan). The target sequences of SPOCK1 shRNA were 5′-CTGCTGGATGACCTAGAATAT-3 and 5′-GCTTTCGAGACGATGATTATT-3. The shRNA lentivirus was produced as previously described [19].

### Plasmid construction and transfection

SPOCK1 Gateway donor complementary (c)DNA was purchased from DNasu Plasmid Repository and then recombined into the plenti6.3-DEST (Invitrogen) vector by Clonase LR (Invitrogen). The Plenti-6.3-SPOCK1, pMD.G, and pCMVDR8.91 plasmids were transfected into 293 T cells for packing the lentivirus. Target cells were incubated with viral supernatants for 48 h.

### Intracardiac experimental metastasis model

PC-3 M-Luc cells were cultured in MEM supplemented with 10% FBS, and API’s curative effects on the progression of established metastases were evaluated as follows. For intracardiac experimental metastasis assays, male NOD-scid IL2Rγ^null^ (NSG) mice (6~7 weeks old) were intraperitoneally (IP) injected with API (3 mg/kg of body weight (BW)) or 10% DMSO 3 days prior to an intracardiac injection and then approximately 10^6^ PC-3 M-Luc cells were inoculated into the left ventricle of the heart by nonsurgical means. Bioluminescence imaging was done 30 min after the intracardiac injection to detect the distribution of PCa cells. Then each treated mouse was administered an IP injection of 3 mg/kg of API 6 days/week for 5 weeks. The injection volume was 100 μL (10 μL of a stock solution and 90 μL of PBS) each day. The control group received 100 μL of vehicle (10 μL of DMSO and 90 μL of PBS). Mice that showed whole-body bioluminescence signals were further monitored with weekly bioluminescence imaging (BLI). Images were acquired and analyzed with an In Vivo Imaging System (IVIS) Spectrum Imaging System (Xenogen, Alameda, CA). *Ex vivo* images of tumor-bearing tissues excised from the mice at necropsy were also obtained. All experiments were conducted in accordance with guidelines and regulations approved by the Institutional Animal Care and Use Committee of Taipei Medical University.

### Orthotopic xenograft mouse model

For SPOCK1 overexpression and knockdown experiments in an orthotopic xenograft mouse model, 5-week-old male NSG mice were anesthetized with pentobarbital; then the PC-3-mock-luciferase, PC-3-SPOCK1-luciferase, PC-3 M-mock-luciferase, or PC-3 M-sh-SPOCK1-luciferase stable cell lines (5 × 10^5^) were resuspended in a 1:1 mixture of PBS and GFR-Matrigel and inoculated into the anterior prostate using a 30-gauge needle, which was inserted through a lower abdominal incision. The incision was closed using a 4–0 Vicryl filament. After 7 days, the mice were randomly assigned to the experimental and control groups according to the Xenogen IVIS spectrum BLI results, such that treatment was initiated at a similar mean tumor size in each group. Then each treated mouse received daily IP injections of 3 mg/kg of API or the vehicle (10% DMSO in PBS) 6 days/week. The day after API treatment, mice were injected with D-luciferin and imaged for 1~2 min using this live imaging device to monitor the tumor size and location in real time. After 35 days, mice were sacrificed, and *ex vivo* images of tumor-bearing tissues excised from the mice at necropsy were further determined using the IVIS-Spectrum system. Tumors were also weighed and fixed, sectioned, and stained with hematoxylin and eosin (H&E) for IHC. All experiments were conducted in accordance with guidelines and regulations approved by the Institutional Animal Care and Use Committee of Taipei Medical University.

### BLI

Mice anesthetized by exposure to 1%~ 3% isoflurane were placed in the IVIS Imaging System (Xenogen) and imaged from ventral views approximately 2 min after an IP injection of 100 μl of d-luciferin (Caliper Life Sciences) at 30 mg/ml per mouse. Established metastasis was assessed by imaging during the treatment period. The acquisition time was 2 min at the beginning of the time course and was progressively reduced afterward in accordance with the signal strength to avoid saturation. The analysis was performed using Living Image software (Xenogen) by measuring the photon flux (photons/s/cm^2^) with a region of interest drawn around the whole-animal or organ bioluminescence signal.

### H&E staining

Tissue sections were routinely de-waxed and rehydrated. Sections were stained in hematoxylin for 5 min, and washed in running tap water for 5 min. Then, sections were stained in eosin for 30 s, dehydrated, and mounted by routine methods. Representative fields were chosen to be presented in the figures.

### IHC analyses

IHC was performed as previously reported [8]. Mouse prostate tissues were fixed in 10% (v/v) formaldehyde in PBS, embedded in paraffin, and cut into 3-μm sections. Prostate sections were deparaffinized in a xylene solution and rehydrated using gradient ethanol concentrations. Deparaffinized sections were boiled in a microwave in 0.1 M citric acid buffer (pH 6.0) for antigen retrieval and then blocked by hydrogen peroxide and blocking serum, followed by incubation overnight with corresponding primary antibody for SPOCK1 (1:200, ab229935) at 4 °C. A secondary antibody was then incubated with the slides at room temperature for 1 h. Sections were observed after diaminobenzidine (DAB) kit (Boster, Wuhan, China) incubation and scored under a light microscope.

### Scoring

The stained SPOCK1 were evaluated with an Olympus BX50 light microscope. For semi-quantitative evaluation of the slides, a multi-score of staining frequency and intensity was applied. The frequency score ranged from 0~5 (negative = 0, < 10% positive cells = 1+, 10%~ 25% = 2+, 25%~ 50% = 3+, 50%~ 75% = 4+, and 75%~ 100% = 5+). The intensity score ranged from 0~3 (weak = 1, moderate = 2, strong = 3). The two results for staining intensity and frequency were multiplied, so that a ‘Multi-Score’ reflected both. Result are scored by multiplying the frequency score by the intensity.

### Statistical analysis

Data are presented as the mean ± standard deviation (SD), and statistical comparisons between groups were made using Student’s *t*-test (two-tailed). Animal survival time was determined utilizing a Kaplan-Meier survival analysis and log-rank test. A *p* value of < 0.05 was considered a statistically significant difference.

## Results

### API treatment results in reduced cell viability and motility of human PCa cells

**Fig. 1:**
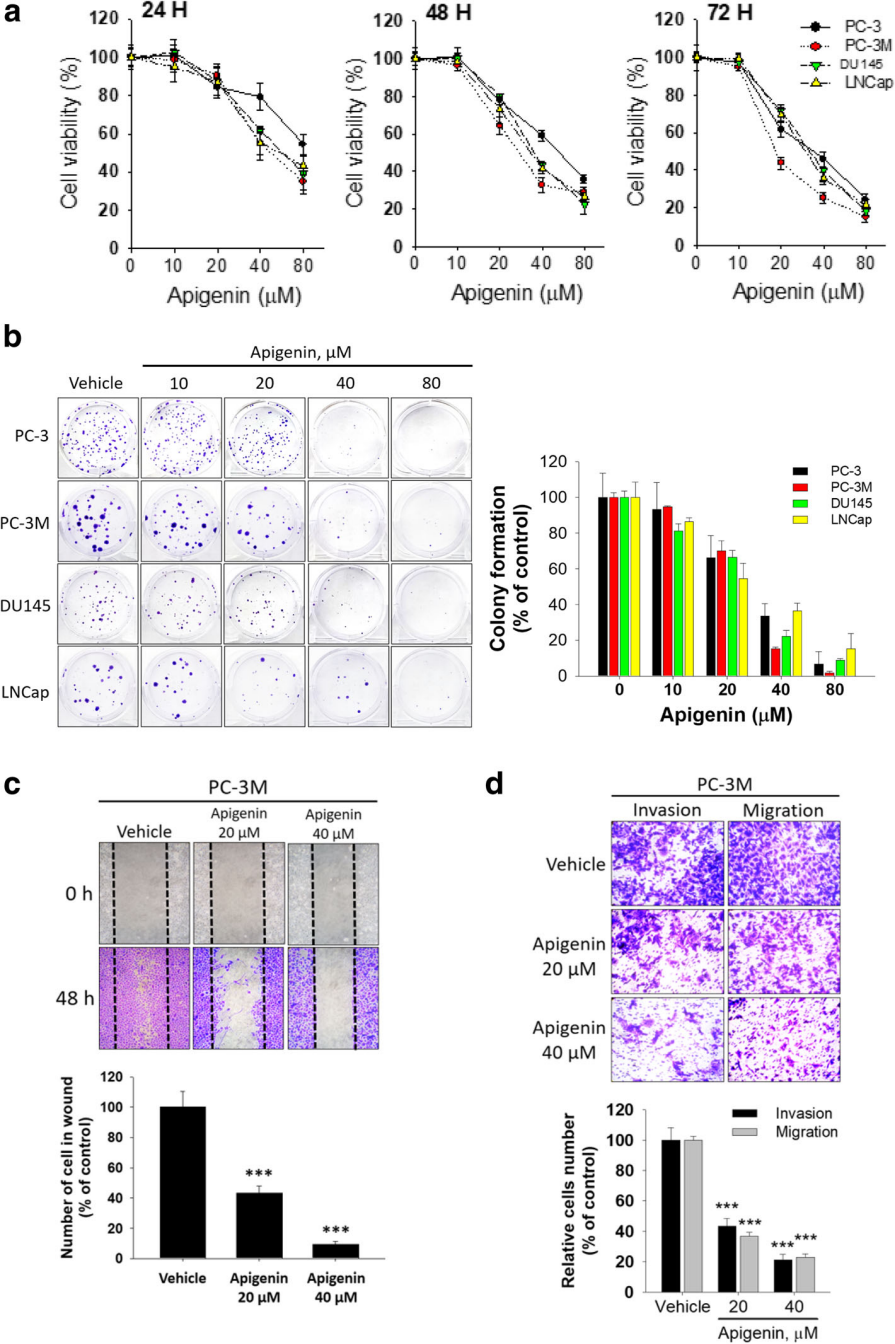
Evaluation of the biologic role of apigenin (API) in prostate cancer cell lines. **a** Four prostate cancer cell lines (LNCap, DU145, PC-3, and PC-3 M) were treated with various doses of apigenin or vehicle (DMSO) for 24, 48, and 72 h. Cell viability was determined by a CellTiter 96® cell proliferation assay. **b** A colony formation assay was performed with the four prostate cancer cell lines in the presence or absence of API. Colonies formed during 14 days of culture were visualized by crystal violet staining (left), and colony numbers were quantified by manual counting (right). **c** Representative images of wounded PC-3 M cells treated with or without 20 or 40 μM API for 48 h. Following incubation, migrated cells were stained with crystal violet (upper panel), and results of the quantitative analysis are expressed as the average number of migrated cells compared to controls (lower panel). **d** Cell migration and invasion determined by a transwell assay. Representative images of cell migration and invasion of PC-3 M cells with or without 20 or 40 μM of API treatment. All values are expressed as the multiple of change relative to the untreated control. Data are presented as the mean ± SD of at three independent experiments. * *p* < 0.05; ** *p* < 0.01; *** *p* < 0.001 compared to the vehicle group

To explore the broad therapeutic potential of API against PCa, we examined the cytotoxic effects of various concentrations of API (approximately 0~80 μM) for 24~72 h on four PCa cell lines (LNCap, DU145, PC-3, and PC-3 M). The MTS assay indicated that API reduced cell survival in time- and dose-dependent manners in all PCa cell lines (Fig. 1a). The IC_50_ values of API were shown in Table 1. Among the four examined PCa cell lines, PC-3 M cells were found to be the most sensitive to API treatment. In addition, long-term growth inhibitory effects of API on PCa cells were further assessed by a colony formation assay. After 14 days, colony numbers of the four prostate cancer cell lines in the API-treated groups were significantly reduced in a dose-dependent manner compared to the control group (Fig. 1b). Subsequently, to assess the biological function of API on cell motility, wound-healing and transwell apparatus assays were performed. In the scratch wound-healing assay, API-treated cells exhibited retarded wound closure, and fewer stained cells had migrated into the wounded area in a dose-dependent manner in PC-3 M (Fig. 1c) and DU145 (Additional file 1: Figure S1). As expected, similar results were obtained in the transwell migration and Matrigel invasion assays of PC-3 M (Fig. 1d) and DU145 cells (Additional file 1: Figure S2). API-treated cells exhibited lower migration and invasion capabilities compared to the control group. Moreover, to further confirm whether the inhibitory effect of API on cell motility because of the API-induced inhibition of cell viability. We normalized the data from wound-healing and transwell migration/invasion assays to the cell viability modulated by API and the results showed that API treatment still significantly suppresses the abilities of wound healing, invasion and migration in PC-3 M and DU145 cells (Additional file 1: Table S1). These findings indicate that API was able to attenuate the aggressive behavior of PCa cell lines.

**Table 1:**
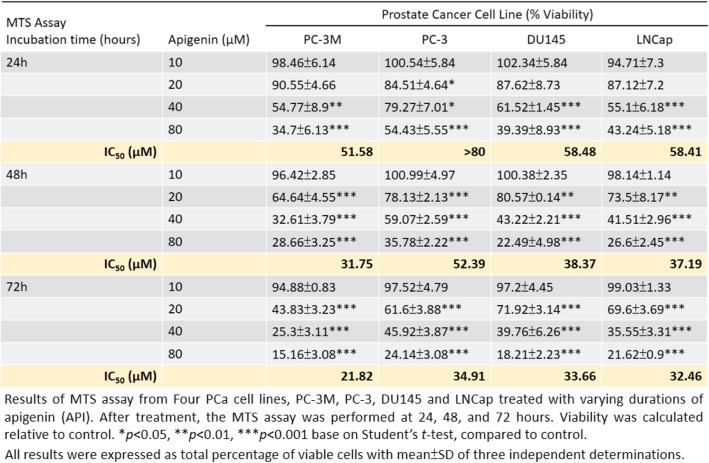
Comparison of cell viability under API treatment in four Different PCa cell lines

### API reverses changes in EMT biomarkers which contributed to suppression of the invasive property of human PCa cell lines

**Fig. 2:**
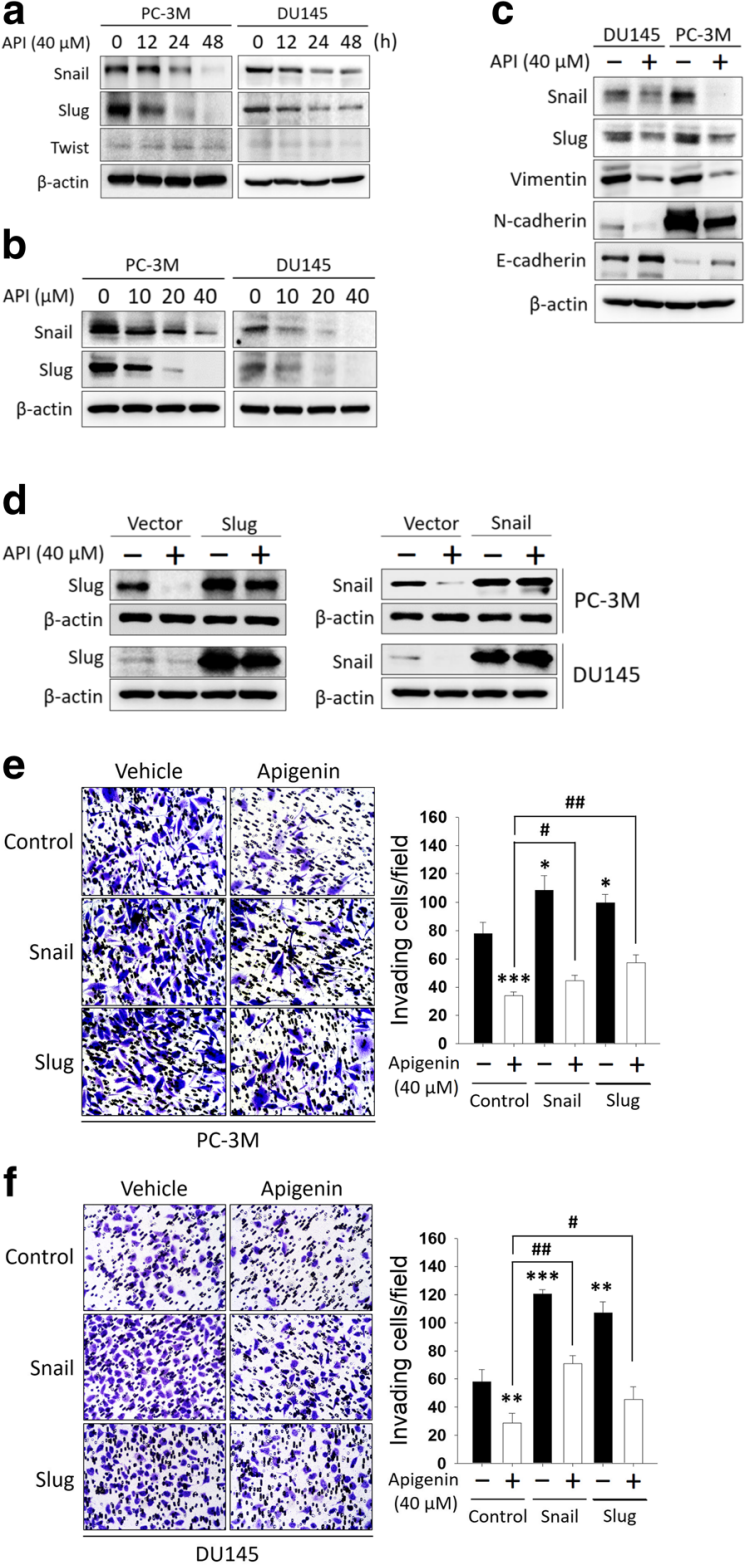
Targeting the Snail/Slug-mediated epithelial-to-mesenchymal transition (EMT) process by apigenin (API) results in suppression of the invasion of prostate cancer cells. **a, b** PC-3 M and DU145 cells were treated with API at 40 μM for different durations (**a)**, or at an increasing dose for 24 h (**b)**, and EMT-related transcription factors (Snail, Slug, and Twist) were evaluated by Western blotting. **c** After API treatment for 24 h, protein expression levels of EMT markers in PC-3 M and DU145 cells were determined by Western blotting. **d** PC-3 M and DU145 cells were transfected with either pLEX-Snail, pCIneo-Slug, or their respective vector controls for 24 h, and then expression levels of Slug (left panel) and Snail (right panel) were examined by Western blotting. **e, f** The vector control or overexpressing cells were incubated with 40 μM API for 48 h, and cell invasive abilities were measured by a transwell assay. Representative photographs of invasive cells (left) and quantification of these cells (right) are shown. Data are presented as the mean ± SD of three independent experiments, * *p* < 0.05, ** *p* < 0.01, *** *p* < 0.001 vs. untreated cells and ^#^ *p* < 0.05, ^##^ *p* < 0.01, ^###^ *p* < 0.001 vs. 40 μM API-treated cells

EMT is a biological process which occurs during tumor progression and metastasis. To characterize the molecular players underlying API’s prevention of PCa aggressiveness in vitro, we first studied whether API blocks PCa cells’ invasive capability by regulating expression profiles of some key transcription factors (TFs) known to promote the EMT. These TFs include the zinc-finger proteins, Snai1 (Snail) and Snai2 (Slug), as well as the basic helix-loop-helix protein, Twist1 (Twist) [20]. PC-3 M and DU145 cells were treated with 40 μM API for indicated time (12, 24, and 48 h) or different doses of API for 24 h, and we found that API reduced Snail and Slug expressions, whereas Twist was not consistently affected (Fig. 2a, b). We further investigated the involvement of downregulation of Snail and Slug expressions in changed expressions of EMT markers, due to API treatment’s contribution to the inhibition of invasion in a PCa cell model. A Western blot analysis demonstrated that API caused reversal of the EMT in PCa cells, as evidenced by increased expression levels of the epithelial marker, E-cadherin, and decreased levels of the mesenchymal markers, vimentin, and N-cadherin (Fig. 2c). To address whether modulation of the Snail/Slug-mediated EMT by API influences the invasive properties of PCa cells, we stably overexpressed Snail and Slug in PC-3 M and DU145 PCa cell lines (Fig. 2d) and demonstrated that overexpression of Snail and Slug caused a significant increase in the invasive ability compared to cells that were transfected with an empty vector. Simultaneously, API-mediated cell invasion inhibitory effects were markedly reversed in Snail-, and Slug-overexpressing cells (Fig. 2e, f). Overall, these results suggested that API impaired the migratory and invasive abilities of PCa cells through altering expressions of EMT markers due to reduced Snail and Slug expressions and subsequently reversing the EMT process.

### API suppresses PCa metastasis and prolongs survival in an *intracardiac injection model*

**Fig. 3:**
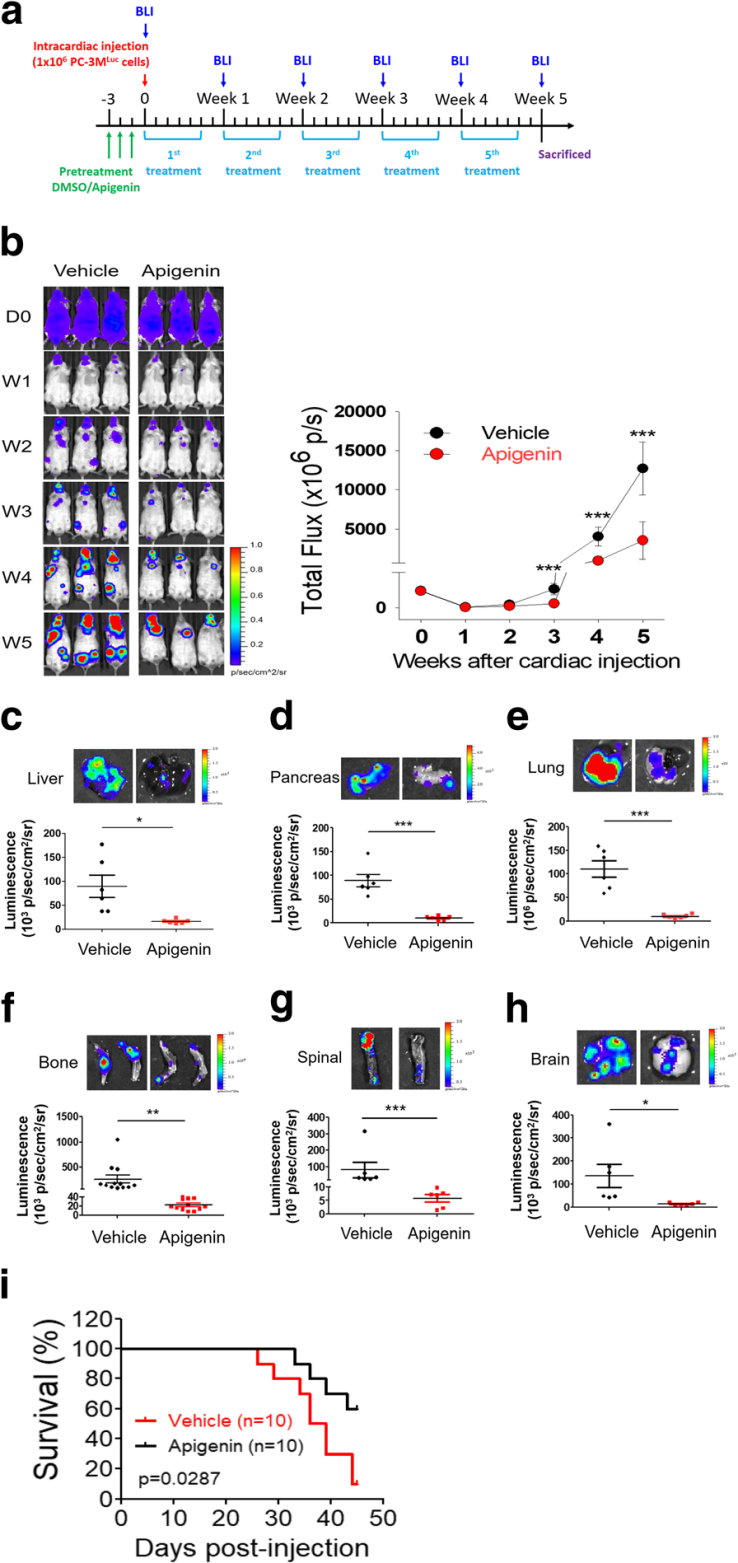
Apigenin (API) reduces multiple metastases and prolongs survival in an experimental metastasis model with an intracardiac injection. **a** Timeline of the in vivo study design for investigating the antimetastatic activities of API. Male NSG mice were intracardially injected with luciferase-tagged PC-3 M cells (PC-3 M-Luc). Mice were pretreated with API (3 mg/kg, IP) or the vehicle for 3 days before the intracardiac injection which was then administered 6 days/week. Whole-body bioluminescent imaging (BLI) was performed at different time points after cell-injection in NSG mice. **b** All mice were sacrificed at 5 weeks after the intracardiac injection of PC-3 M-Luc cells, and luciferase activity was detected every week with an IVIS spectrum imaging system (right panel). Quantitative analysis of Xenogen imaging signal intensity (photons/s) every week (left panel). * *p* < 0.05, ** *p* < 0.01, *** *p* < 0.001 compared to the control group. **c-h** *Ex vivo* representative images of metastatic sites at the end of this metastasis assay in mice treated with API (3 mg/kg, IP) or the vehicle (upper panel). Liver (**c**), pancreas (**d**), lungs (**e**), bone (**f**), spinal cord (**g**), and brain (**h**) are major organs for metastasis. Signal intensities from these metastatic organs were bioluminescently captured at the end of the study, with the mean signal for each group indicated (lower panel). * *p* < 0.05, ** *p* < 0.01, *** *p* < 0.001 compared to the control group. **i** Kaplan-Meier survival curves for control and apigenin (3 mg/kg)-treated mice. *p* values were analyzed by the log-rank test

To rule out the possibility that API’s inhibitory effect on cell invasion was because of API-mediated growth inhibition, we further investigated the effect of API on the homing capacity and growth in metastatic organs using an intracardiac injection model with a BLI analysis, which mimics the clinical situation of cancer metastases to distant sites. A schematic timeline of this experimental design and setup is shown in Fig. 3a. We established an intracardiac PCa metastasis model by stable transfection with firefly luciferase to generate the PC-3 M-Luc cell line to allow the in vivo monitoring and quantification of BLI of tumor cells. Mice were given an IP injection of API (3 mg/kg of BW) or 10% DMSO 3 days prior to the intracardiac injection, and then a successful intracardiac injection was indicated on day 0 by images showing systemic bioluminescence distributed throughout the animal. After the PC-3 M-Luc cell injection, mice were given an IP injection of API or 10% DMSO six times per week for 5 weeks. Within 2 weeks after the intracardiac injections began, metastasis bioluminescence was observed in the head, chest, and knee of the control group, while treatment with API inhibited luminescence signaling, and no metastasis bioluminescence was observed in the knee. At 3 weeks post-injection, mice exhibited clear signs of distant metastatic lesions by luciferase imaging in the control group; however, API-treated mice manifested a significant decrease in established metastatic sites and visibly reduced bioluminescent signals (Fig. 3b). To visualize the organs affected by metastases, mice were sacrificed at 5 weeks post-injection, and *ex vivo* imaging evidenced metastasis to the lungs, liver, pancreas, spine, bone, and brain, and treatment with API reduced luminescence signaling and metastatic sites (Fig. 3c-h). Mice survival curves were generated from the first API administration, followed by the 5-week-treatment. Under this schedule, treatment of tumor-bearing mice with API led to a significant increase in the survival rate compared to that of control tumor-bearing mice (Fig. 3i). Collectively, these results provide evidence that API reduced PCa metastasis in vivo and seemed to have a beneficial effect of prolonging survival.

### API inhibits PCa cells invasion through modulation of the SPOCK1-mediated EMT process

**Fig. 4:**
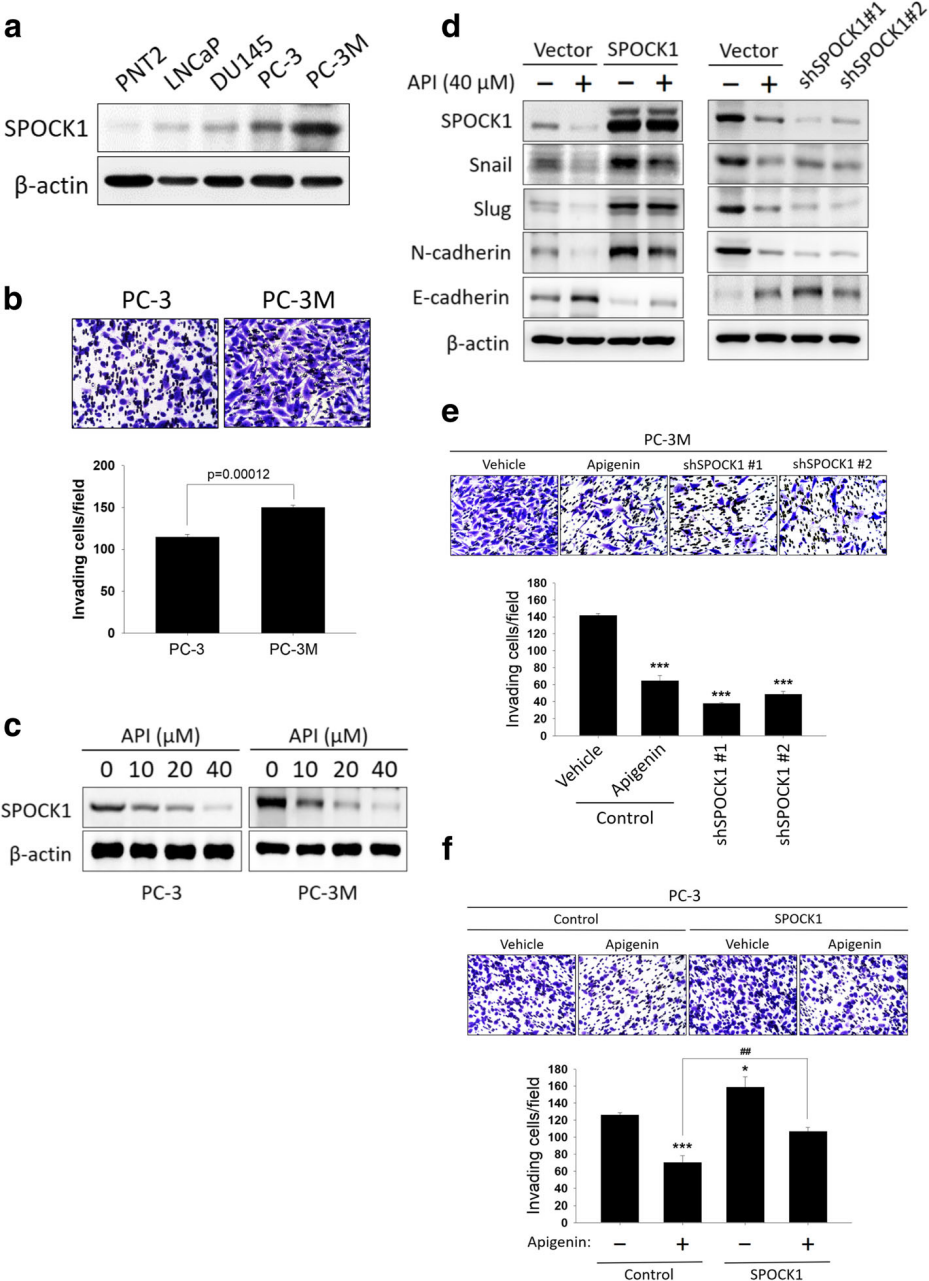
Interplay between SPOCK1 and epithelial-to-mesenchymal transition (EMT) marker expressions is involved in apigenin (API)-mediated inhibition of cell motility. **a** Western blot analysis of the protein level of SPOCK1 in PNT2 prostate epithelial cells and LNCaP, DU145, PC-3, and PC-3 M prostate cancer cell lines. The protein level of SPOCK1 was higher in PC-3 M cells. **b** A Matrigel invasion assay was performed to study the invasive abilities of PC-3 and PC-3 M cells. Quantitative results of counting invaded cells in a 200× field. **c** SPOCK1 expression was assessed by a Western blot analysis in PC-3 and PC-3 M cells after treatment with various concentrations of API for 24 h. **d-f** Influences of SPOCK1 on EMT-related marker expressions (**d**) and invasive abilities of PC-3 M (**e**) and PC-3 cells (**f**) in response to API

SPOCK1 exerts oncogenic activities in human PCa tumorigenesis and metastasis [8]. In order to determine whether API interferes with cell invasion by modulating SPOCK1 expression in PCa cells, we first evaluated endogenous protein expression levels of SPOCK1 in prostate epithelial PNT2 cells, the weakly malignant LNCaP cells, and the highly malignant DU145, PC3, and PC-3 M cells. PC-3 M cells are a metastasis-derived variant of PC-3 cells, which are more highly metastatic than PC-3 cells [21]. Results showed that SPOCK1 was barely detectable in PNT2 cells and SPOCK1 expression seemed to correlate the malignancy of PCa cells (Fig. 4a). Strikingly, compared to PC-3 cells, the metastatic variant PC-3 M cells expressed higher levels of the SPOCK1 protein (Fig. 4a) and exhibited more-invasive properties (Fig. 4b). From these results, PC-3 and PC-3 M cell lines were selected for subsequent in vitro and in vivo studies to identify the possibility mechanisms of SPOCK1 in PCa progression. Treatment of PC-3 and PC-3 M cells with different concentrations of API significantly reduced SPOCK1 expression (Fig. 4c). Additionally, we overexpressed SPOCK1 in PC-3 cells and employed specific shRNA to deplete SPOCK1 expression in PC-3 M cells to confirm whether SPOCK1 is involved in API-mediated suppression of PCa invasive abilities. Results showed that the SPOCK1 protein level increased by transfection of its expression plasmid in PC-3 cells, while it decreased in response to its specific shRNA in PC-3 M cells (Fig. 4d). Moreover, we found that SPOCK1 shRNA was similar to treatment with API in PC-3 and PC-3 M cells, which led to reduced Snail and Slug expressions, and then decreases in levels of the mesenchymal proteins, N-cadherin, and increased expression levels of the epithelial marker, E-cadherin, whereas these were reversed in PC-3 cells with stable transfection of SPOCK1 (Fig. 4d). Furthermore, API and shRNA transfection efficiently attenuated the invasive capacities of PC-3 M cells (Fig. 4e), and overexpression of SPOCK1 restored the repressive effect of API in PC-3 (Fig. 4f), LNCaP, and DU145 cells (Additional file 1: Figure S3a, b). Taken together, these observations suggest that suppressing SPOCK1 is crucial for API-mediated inhibition of EMT progression and cell invasion in PCa cells.

### Targeting SPOCK1 with API suppresses orthotopic prostate tumor growth and spontaneous metastasis

**Fig. 5:**
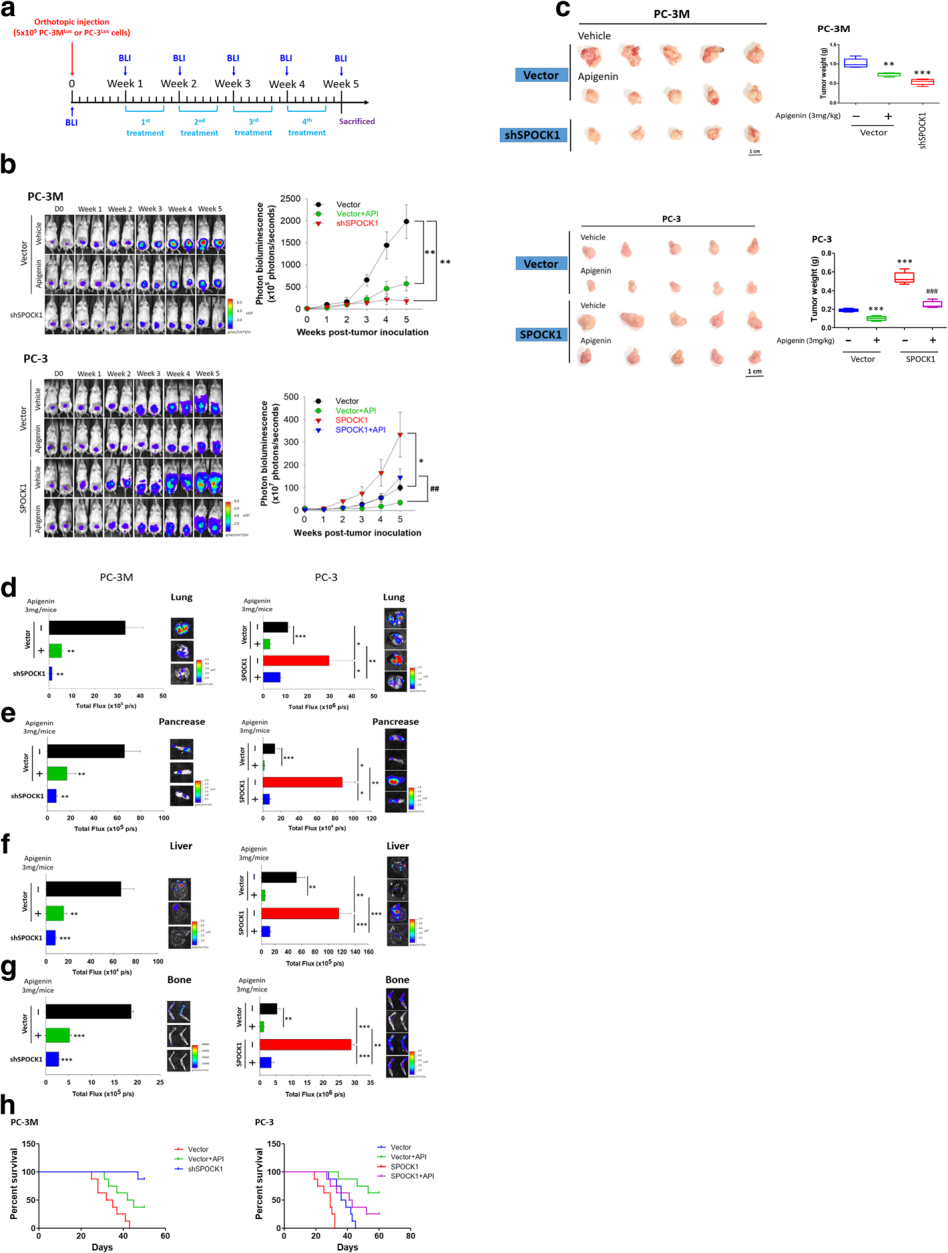
Targeting SPOCK1 suppresses prostate cancer growth and metastasis by apigenin (API) in an orthotopic mouse model. **a** Timeline of the in vivo study design for investigating the effects of SPOCK1 expression on tumor progression and the antitumor activity of API. Male NSG mice were orthotopically injected with luciferase-tagged and SPOCK1-depleted (shSPOCK1) PC-3 M cells or SPOCK1-overexpressing PC-3 cells. After 7 days, mice were treated with API (3 mg/kg, IP) or the vehicle for 6 days/week. Whole body bioluminescence imaging was conducted at different time points after cell-injection in mice. **b** All mice were sacrificed and dissected at 4 weeks after API treatment, and the luciferase activity was detected every week with an IVIS imaging system (left panel). Quantitative analysis of the Xenogen imaging signal intensity (photons/s) every week (right panel). **c** Tumors were dissected and photographed after 5 weeks (left panel), and the average tumor weight in each group is given (right panel). Data are presented as the mean ± SD. * *p* < 0.05, ** *p* < 0.01, *** *p* < 0.001 compared to the control group. ^#^ *p* < 0.05, ^##^ *p* < 0.01, ^###^ *p* < 0.001 compared to the API-treated group. **d-g** Representative *ex vivo* bioluminescence imaging of metastatic sites at the end of this spontaneous metastasis assay. Lungs (**d**), pancreas (**e**), liver (**f**), and bone (**g**) are major organs for metastasis, and signal intensities of metastatic organs were imaged with bioluminescence at the end of the study, with the mean signal for each group indicated. Data are presented as the mean ± SD. * *p* < 0.05, ** *p* < 0.01, *** *p* < 0.001. **h** Kaplan-Meier curves and log-rank test of overall survival analysis for indicated tumor-bearing mice treated with API (3 mg/kg, IP) or the vehicle

To explore whether SPOCK1 is an effective therapeutic target of API in PCa, we tested the role of SPOCK1 in tumor metastasis and the antimetastatic activity of API in vivo using stable SPOCK1-silenced PC-3 M-Luc cells or SPOCK1-overexpressing PC-3-Luc cells. To investigate the efficacy of the targeting of SPOCK1 by API on the prostate tumor growth and metastasis cascade, we established a murine orthotopic xenograft model and monitored tumor metastasis via a non-invasive IVIS. In total, 5 × 10^5^ cells of various cloned cell lines were inoculated into the prostate of NSG mice, and tumor progressions were evaluated by IVIS. A schematic timeline of this experimental design and setup is shown in Fig. 5a. In agreement with the in vitro results, bioluminescence signals of tumors had decreased in mice that received IP injections of API (3 mg/kg), compared to mice injected with 10% DMSO in both the PC-3 and PC-3 M groups, and similar results were observed in the stable SPOCK1-depleted (shSPOCK1) PC-3 M group, whereas the stable SPOCK1-overexpressing PC-3 group exhibited a much higher photon intensity which was sufficient to reverse the bioluminescent signals of API treatment (Fig. 5b). Moreover, the tumorigenic ability was reduced with API treatment and SPOCK1-targeted shRNA compared to mock-transfected tumors; conversely, in SPOCK1-overexpressing tumors, the tumor growth was more rapid and significantly reversed the antitumor effects of API as evidenced by the tumor size and weight (Fig. 5c). In addition, to avoid the possibility that metastasis were missed because of inappropriate imaging, we conducted *ex vivo* imaging. All mice were sacrificed at 5 weeks after inoculation with various clones of cancer cells, and organs were dissected to visualize organ metastases by BLI. From the *ex vivo* imaging of different tissues, we also observed that SPOCK1-depleted or API treatment significantly abated the multiorgan metastatic potential of PCa, and this phenomenon was reversed in PC-3-Luc cells overexpressing SPOCK1 (Fig. 5d-g). In an overall survival analysis, a parallel study in a different animal cohort was also carried out and showed that mouse survival in the model was inversely proportional to SPOCK1 expression. Results demonstrated that mice with shSPOCK1-PC-3 M-Luc tumors or API treatment had significantly prolonged survival compared to mice with vehicle treatment, and those overexpressing SPOCK1 had shorter overall survival (Fig. 5h). These data suggested that API disrupts PCa growth and metastasis by suppressing the SPOCK1 expression and indicated that SPOCK1 may be an effective therapeutic target in PCa.

### Suppression of SPOCK1 protein expression by API contributes to inhibition of EMT activation in vivo

**Fig. 6:**
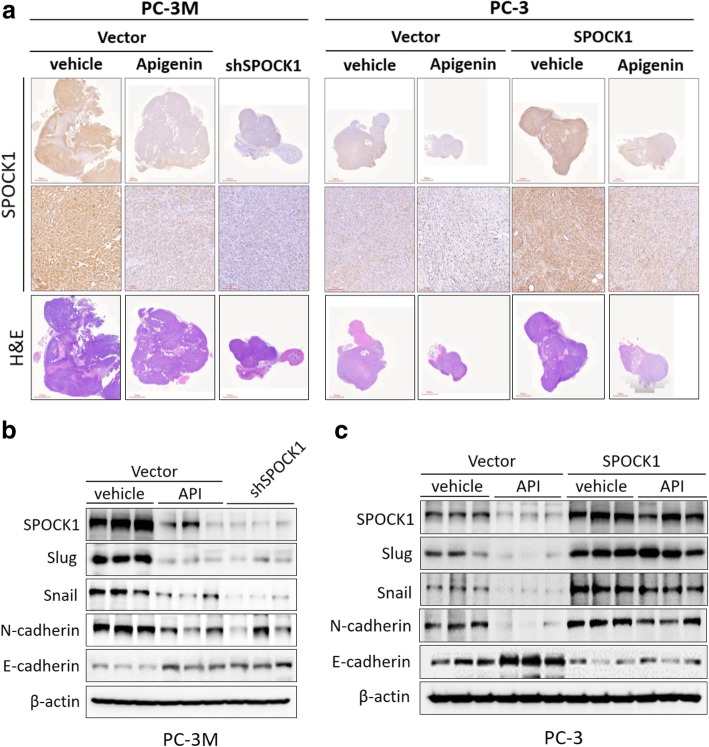
Modulation of SPOCK1 by apigenin (API) interrupted epithelial-to-mesenchymal transition (EMT) activation in vivo. Representative examples of tumors formed in mice injected with the indicated cells**. a** Histological and IHC analysis of sections from tumor samples for SPOCK1. **b, c** Western blot analysis of expressions of SPOCK1 and a series of EMT-related proteins in tumor tissues of the orthotopic prostate cancer model

To confirm the role of SPOCK1 in anti-tumorigenesis activity of API by IHC staining, we found that SPOCK1 staining was markedly decreased in shSPOCK1 and API treatment, whereas SPOCK1-overexpressing tumor tissues were strongly stained, and the suppressive effects of API had been restored (Fig. 6a). Our data imply that SPOCK1 expression mediates EMT signaling activation in vitro, thus, we further detected SPOCK1 and EMT biomarkers by Western blotting in mice bearing tumor xenografts. Consistently, results from the Western blot analysis of tumor tissues indicated that mesenchymal markers (Snail, Slug and N-cadherin) were downregulated while epithelial marker (E-cadherin) was upregulated following SPOCK1 depletion and API treatment but upregulated E-cadherin, an epithelial marker in. Inversely, tumors derived from SPOCK1 overexpression of PC-3 cells reversed this phenomenon (Fig. 6b, c). These findings suggested that the anti-tumorigenesis activities of API in vivo were associated with the downregulation of SPOCK1 expression in human PCa, and were most likely mediated through suppression of the SPOCK1-mediated EMT signaling pathway.

### SPOCK1 was overexpressed in PCa and correlated with poor survival

**Fig. 7:**
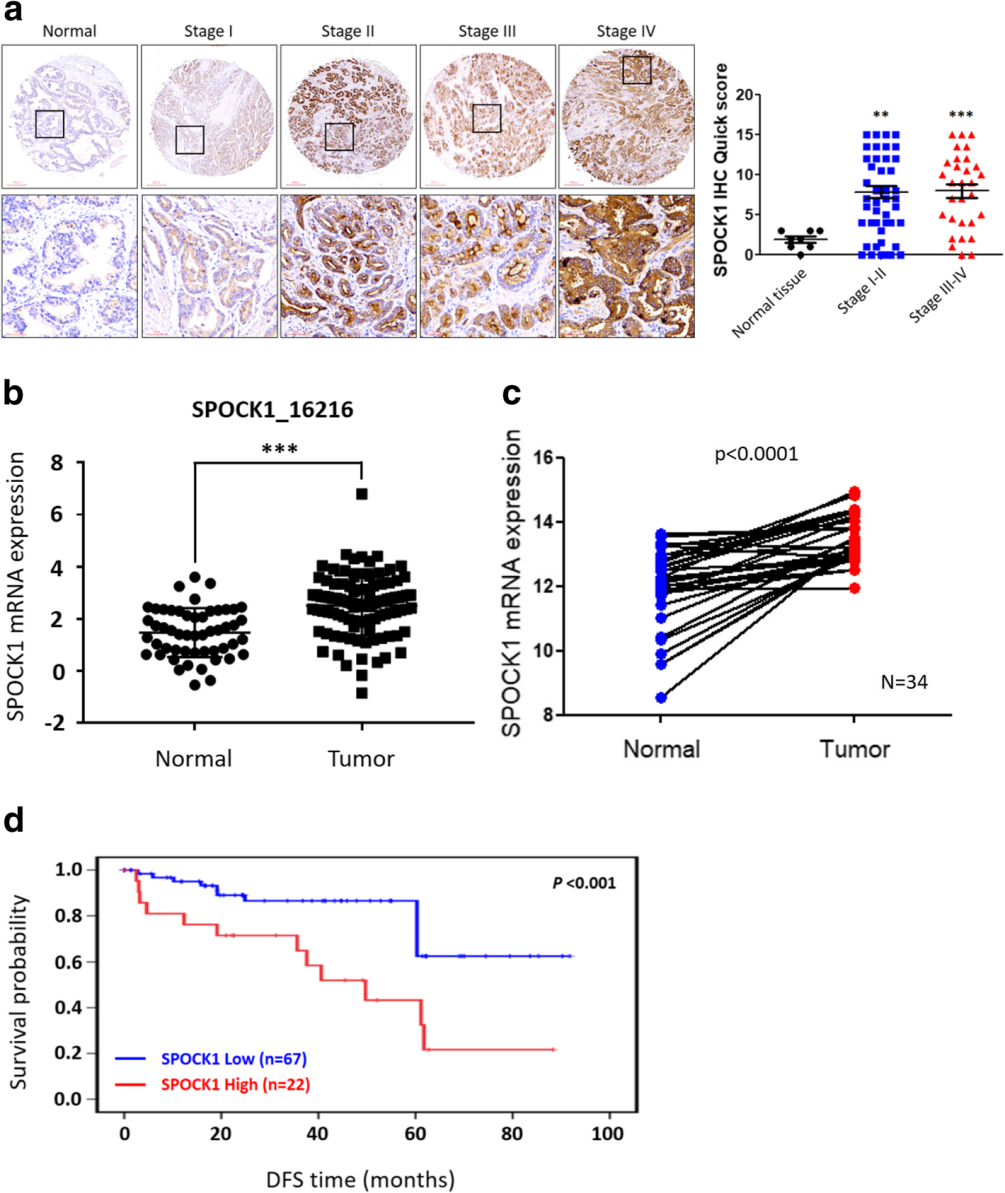
Clinical significance of SPOCK1 in human prostate cancer (PCa). **a** Left panel: Representative immunohistochemical (IHC) staining for SPOCK1 in a prostate cancer tissue microarray (Biomax.US, PR1921a). Right panel: Comparison of IHC staining of SPOCK1 in normal prostate tissues and prostate cancer tissues using IHC scores. SPOCK1 expression was significantly higher in PCa tissues than in normal prostate tissues. Black boxes indicate the enlarged area. **b** Expression of SPOCK1 was assessed in prostate adenocarcinoma patients from GEO database (GSE40272). **c** SPOCK1 expression was significantly higher in PCa tissues compared to paired normal prostatic tissues from TCGA database. **d** Kaplan-Meier analysis of *SPOCK1* gene expression in the GSE40272 human prostate cancer cohort (*n* = 89)

To better evaluate SPOCK1 expression in PCa tissues, we examined SPOCK1 expression by IHC in 96 cases in a PCa tissue array (PR1921a). In normal prostate tissues, SPOCK1 generally displayed very light staining, whereas SPOCK1 was moderately and/or strongly positive stained in cancer samples. Statistical analyses of the IHC staining revealed that SPOCK1 expression produced significantly higher immunoreaction scores in PCa tissues compared to those in normal prostate tissues (Fig. 7a). Additionally, to further elucidate the clinical relevance of SPOCK1, a cohort of PCa cases from The Cancer Genome Atlas (TCGA) and Gene Expression Omnibus (GEO) were analyzed. Of the GEO dataset (GSE40272), SPOCK1 was found to exhibit significantly higher expression levels in PCa tissues compared to normal prostate tissues (Fig. 7b). On the other hand, in the TCGA dataset, we further confirm the significance of SPOCK1 in PCa progression, we collected 34 cases of primary tumors with matched non-tumor tissues from the cohort and found that SPOCK1 expression was also significantly higher in the tumor tissues compared to that in their paired normal tissues (Fig. 7c). In addition, disease-free survival (DFS) curves of all patients were computed with the Kaplan-Meier method and compared between groups using the log-rank test; results showed that patients with tumors exhibiting high expression levels of SPOCK1 had significantly shorter DFS times compared to patients with tumors exhibiting low SPOCK1 expression levels (Fig. 7d). These results suggested that aberrantly higher expression of SPOCK1 had crucial effects on the poor prognosis of patients with PCa.

## Discussion

PCa is one of the leading causes of cancer-related deaths in men worldwide, and metastasis is the primary factor in PCa mortality. Until recently, the mainstay of therapy for patients with metastatic PCa principally focused on targeting the androgen receptor (AR) [22, 23]. Thus, ADT is universally accepted as the initial treatment for men with locally advanced and metastatic PCa, which reduces levels of androgen hormones and is accomplished by luteinizing hormone-releasing hormone (LHRH) agonist drugs or surgical castration [24]. Unfortunately, as demonstrated in nearly all patients receiving ADT, it ultimately results in relapse and development of clinically androgen-independent PCa (AIPC), and tumor progression inevitably occurs [25]. This suggests that the high rate of mortality from PCa is linked to the development of AIPC and the current lack of effective therapies. Therefore, developing novel therapeutic approaches to target AIPC has considerable potential for improving the quality of life and survival of patients with metastatic PCa. Our present results showed that API significantly inhibited the cell viability of both androgen-independent DU145, PC3, and PC-3 M cells and the androgen-dependent LNCaP human PCa cell lines. Moreover, our results showed that API suppressed the invasive ability of androgen-independent DU145, PC3, and PC-3 M cells, which were respectively derived from brain, bone, and liver metastasis, and possess high tumorigenic and metastatic capacities. Our studies also identified that SPOCK1 plays an important role in regulating the invasive abilities of several AIPC cell lines (PC3 and PC-3 M) and can be downregulated by API treatment in those cell lines. Importantly, for the first time we demonstrated that the antimetastatic effect of API is due to suppression of SPOCK1-mediated EMT activation in a human PCa xenograft model. These results suggest that suppression of SPOCK1 might be a general phenomenon in API-regulated cell motility of PCa cells.

Metastasis is a multistep cellular process, and most PCa-related deaths are due to metastatic disease rather than due to the corresponding primary tumors. Strikingly, the EMT is a crucial step in tumor progression and plays a pivotal role during cancer invasion and metastasis [26]. SPOCK1 encodes a matricellular glycoprotein belonging to the secreted protein, acidic, cysteine-rich (SPARC) family, which consists of SPARC, Hevin, testican-2, testican-3, and follistatin-like protein 1. Members of this protein family act to modulate interactions of extracellular proteins with cell surfaces to direct factors to their suitable extracellular sites [27]. Clinical association studies found that SPOCK1 expression in metastatic tissues was significantly higher than in non-metastatic cancerous tissues [8]. Similar to previous findings, we also observed that SPOCK1 was expressed more strongly in PCa tissues relative to noncancerous tissues, and a high SPOCK1 expression level was significantly correlated with worse disease-free survival compared to PCa patients with lower levels. Additionally, in vivo studies of lung cancer demonstrated that SPOCK1 is not only associated with metastasis but also induces the EMT [28], suggesting the extensive roles of SPOCK1 in promoting cancer cell invasiveness and metastasis through involvement in the EMT process [5, 29, 30]. Our results showed lower expression of SPOCK1 in PC-3 cells than the isogenic but metastatic variant PC-3 M cells. Moreover, transcription factors of the Snail family (Snail and Slug) were associated with EMT progression during PCa metastasis [31]. Notably, our present study first demonstrated that API treatment attenuated growth and metastasis of PCa through inactivation of the Snail/Slug-dependent EMT process by suppressing SPOCK1 expression.

Matrix metalloproteinases (MMPs) are a family of zinc-dependent endoproteinases, that directly degrade components of the extracellular matrix (ECM), an important proteolytic event in the invasion and metastasis of tumors [32]. Several clinical assays have confirmed the MMP expression is tied to tumor aggressiveness, disease progression, and clinical outcomes in patients with various types of tumors [33, 34]. Of note, MMP-3 and MMP-9 are detected as mesenchymal markers that stimulate the EMT process and contribute to metastasis [35, 36]. On the other hand, transcription factors of the Snail family (Snail and Slug), known to regulate EMT pathways, were also shown to regulate several MMPs [37, 38]. This evidence suggests that cooperation and crosstalk between MMPs and EMT-associated transcription factors may be involved in the process of SPOCK1 inducing the EMT. Indeed, a recent investigation revealed that SPOCK1-promoted tumor growth and metastasis are accompanied by upregulation of MMP-3 and MMP-9 expressions in PCa [8], suggesting that MMPs may be involved in the SPOCK1-mediated EMT process and subsequent metastasis. The antimetastatic properties of API are that it inhibits cancer cell migration and invasion through attenuation of MMP-9 expression in vitro and in vivo [39, 40]. Our recently published report indicated that API also suppressed CD26 expressions and the EMT-mediated cell invasion in several NSCLC cell lines [16]. In this study, we founded that API treatment of PCa cells caused a decrease in SPOCK1 expression subsequently leading to inhibition of the invasive abilities and EMT-related markers (Snail, Slug and vimentin), while overexpression of SPOCK1 could reverse the API-mediated inhibitory effects, suggesting that SPOCK1 inhibition by API may be the main cause for the API-mediated suppression of Snail family-induced cell motility in PCa.

The PI3K/Akt signaling pathway has been linked to the apoptosis, autophagy, and tumor development, growth, and metastasis. Aberrant expression and activity of the PI3K-Akt pathway was shown to be more frequently observed as PCa progresses toward therapeutic-resistant or metastatic disease [41], suggesting that this pathway may be implicated in the aggressive phenotype of PCa. Accumulating evidence has demonstrated that activation of the PI3K/Akt pathway can upregulate expressions of Snail and Slug by targeting glycogen synthase kinase (GSK)-3β-mediated degradation, thereby triggering the EMT process [42]. On the other hand, activation of the PI3K/Akt pathway was also reported to play a role in cancer cell invasion and metastasis through increasing MMP production via its multiple downstream target proteins such as mammalian target of rapamycin (mTOR), nuclear factor (NF)-κB, GSK-3, and activator protein (AP)-1 [43–45]. Recently, it is worth noting that knockdown of SPOCK1 obviously significantly decreased levels of PI3K and Akt phosphorylation in PCa cells [8]. Moreover, SPOCK1 was able to block apoptosis and promote proliferation, invasion, and metastasis in vitro and in vivo through activating PI3K/Akt signaling in various human malignancies [7, 8, 29]. According to recent literature data, we noted that API can directly block the ATP-binding site of PI3K resulting in suppression of PI3K activity and subsequent inhibition of Akt kinase activity [46]. Additionally, API was observed to exhibit chemopreventive and/or anti-carcinogenic properties in many different cancer cell types due to the inhibition of PI3K/Akt, mitogen-activated protein kinase (MAPK), and NF-κB signaling pathways [46, 47]. Actually, our current results showed that treatment of SPOCK1-overexpressed DU145 and PC-3 cells with a PI3K inhibitor, LY294002, or API all can reverse SPOCK1-induced increases of Akt activation and Snail/Slug expression. (Additional file 1: Figure S4a). Moreover, treatment of PC-3 M cells with LY294002 showed the similar inhibitory effect with API treatment or SPOCK1 knockdown on Akt activation and Snail/Slug expression. (Additional file 1: Figure S4b). Taken together, these data suggest that PI3K/Akt pathway participates in the SPOCK1-regulated Snail family expression and EMT progression in PCa cells and SPOCK1-Akt-Snail/Slug signaling pathway could be a critical target of API to suppress metastasis of PCa. In addition, whether the SPOCK1-Akt-MMP pathway might be another target of API to control the invasive ability of PCa should be further evaluated in the future.

Despite ADT remaining the principal treatment for PCa patients with locally advanced and metastatic disease, most patients exhibit ADT failure and progress to CRPC, leading to their death within a few years. Of note, inhibition of the PI3K pathway causes increased androgen receptor (AR) protein levels and AR target gene expression [48, 49]. Conversely, the PI3K/Akt pathway can suppress AR transcriptional activity and thus contributes to castration resistance, suggesting that this pathway is implicated in the progression to castration resistance [50]. Moreover, androgen-dependent LNCaP cells grown in androgen-depleted medium developed androgen-independent growth and high levels of Akt activation. Similarly, Akt activation also increased in LNCaP xenografts grown in castrated mice [51]. Accordingly, the tumor suppressor, phosphatase and tensin homolog (PTEN), is a lipid phosphatase that restrains PI3K/Akt signaling, and loss of PTEN protein expression was associated with Akt hyperactivation. Subsequent experiments in mice with prostate PTEN deletion (Pten^loxp/loxp^) clearly demonstrated that castrate-resistant cancer developed in regions of the prostate with simultaneous loss of AR expression [50]. Recently, overexpression of SPOCK1 was observed in CRPC tissues [52], suggesting that SPOCK1 activates PI3K/Akt signaling [7, 8, 29], which may partially be a potential mechanism for CRPC development. Moreover, in CRPC cells, the EMT is associated with metastatic processes and leads to drug resistance [53]. Despite many recent advances in PCa therapies, the ultimate development of metastatic (m)CRPC with recurrence to lethal disease remains incurable. Our study demonstrated that API inhibits cell invasion and metastasis through suppressing the SPOCK1-mediated EMT process in mCRPC cell lines (PC3, PC3M, and DU145). Collectively, it was interesting to note that cooperation and crosstalk between SPOCK1-induced PI3K/Akt signaling and MMPs may be involved in PCa progression, and the effects of API on these processes in mCRPC should be further evaluated in the future. Finally, our findings reinforced the notion of API as a dietary supplement or potential therapeutic agent for managing mCRPC.

## Conclusion

**Fig. 8:**
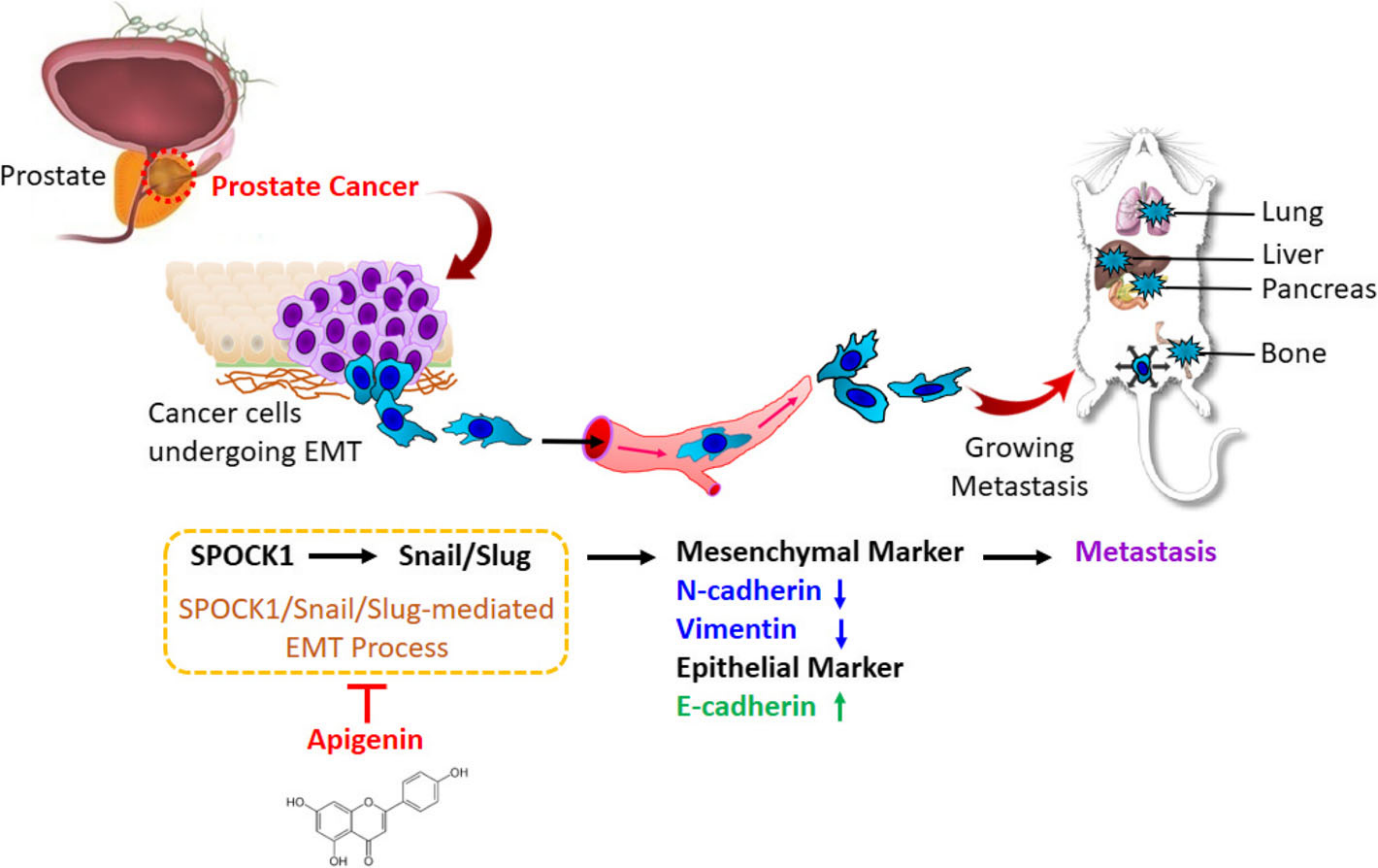
Schematic illustration of the molecular mechanism underlying the ability of apigenin (API) to suppress prostate cancer metastasis. The metastasis-suppressive effect of API on prostate cancer cells was attributed to attenuation of SPOCK1-mediated Snail/Slug expression which resulted in ultimate restraint of EMT progression and subsequent suppression of prostate cancer metastasis

In summary, we characterized and functionally analyzed SPOCK1 in PCa response to API. Our findings demonstrated the importance of increased SPOCK1 expression as a common event, a prognostic indicator of PCa tumorigenesis, and a potential link to tumor metastasis. SPOCK1 may functionally play a critical role in API-regulated antiproliferative and antimetastatic activities in PCa both in vitro and in vivo. Our study defines a mechanism for the suppressor function of API that directly targets SPOCK1, and in turn, inhibits PCa metastasis through inactivating Snail/Slug-mediated EMT activation (Fig. 8). These results suggest that API may be a potential therapeutic agent for PCa treatment, and SPOCK1 may serve as a novel prognostic biomarker and a potential antimetastatic therapeutic target.

## Additional files

Additional file 1: **Figure S1.** Wound-healing assay of apigenin (API)-treated DU145 prostate cancer cells. **Figure S2.** Migration and Matrigel invasion assay of DU145 cells using a Transwell system. **Figure S3.** SPOCK1 is critical for apigenin (API)-modulated invasiveness of prostate cancer cells. **Figure S4.** Akt is a downstream regulator of SPOCK1 to regulate Snail family expression in apigenin (API)-treated prostate cancer cells. **Table S1.** Normalization of wound healing, cell invasion and migration to cell viability under API treatment. (DOCX 1043 kb)

## Data Availability

All data generated or analyzed during this study are included in this published article.

## Abbreviations

ADT: Androgen deprivation therapy
AIPC: Androgen-independent PCa
AP-1: Activator protein-1
API: Apigenin
AR: Androgen receptor
CRPC: Castration resistant prostate cancer
ECM: Extracellular matrix
EMT: Epithelial to mesenchymal transition
GEO: Gene Expression Omnibus
GSK-3β: Glycogen synthase kinase 3β
IHC: Immunohistochemistry
LHRH: Luteinizing hormone-releasing hormone
MMPs: Matrix metalloproteinases
PCa: Prostate cancer
PTEN: Phosphatase and tensin homolog
SPOCK1: Sparc/osteonectin, cwcv, and kazal-like domains proteoglycan 1
TCGA: The Cancer Genome Atlas
