# Supplementary material for: Targeting the SPOCK1-snail/slug axis-mediated epithelial-to-mesenchymal transition by apigenin contributes to repression of prostate cancer metastasis
Source: J Exp Clin Cancer Res. 2019 Jun 10;38:246. doi: 10.1186/s13046-019-1247-3 (PMC6558790; doi:10.1186/s13046-019-1247-3)

**Supplemental Material**

**Title:**

**Targeting the SPOCK1-Snail/Slug axis-mediated epithelial-to-mesenchymal transition by apigenin contributes to repression of prostate cancer metastasis**

Ming-Hsien Chien^†^, Yung-Wei Lin^†^, Yu-Ching Wen, Yi-Chieh Yang, Michael Hsiao, Junn-Liang Chang, Hsiang-Ching Huang, Wei-Jiunn Lee*

Correspondence to: Dr. Wei-Jiunn Lee (E-mail: lwj5905@gmail.com)

**Figure Legends**


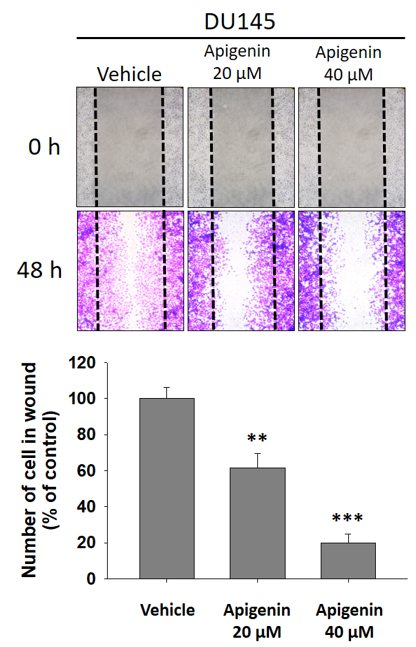


**Additional file 1: Figure S1.** Wound-healing assay of apigenin (API)-treated DU145 prostate cancer cells. Representative images of wounded DU145 cells treated with or without 20 or 40 μM API for 48 h. Following incubation, migrated cells were stained with crystal violet (upper panel), and results of the quantitative analysis are expressed as the average number of migrated cells compared to controls (lower panel). Data are presented as the mean ± SD of at three independent experiments. * *p*<0.05; ** *p*<0.01; *** *p*<0.001 compared to the vehicle group.


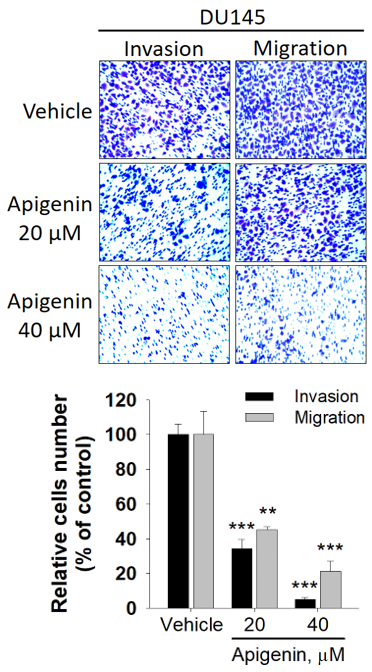


**Additional file 1: Figure S2.** Migration and Matrigel invasion assay of DU145 cells using a Transwell system. Representative images of cell migration and invasion of DU145 cells with or without 20 or 40 μM of API treatment. All values are expressed as the multiple of change relative to the untreated control. Data are presented as the mean ± SD of at three independent experiments. * *p*<0.05; ** *p*<0.01; *** *p*<0.001 compared to the vehicle group.

**
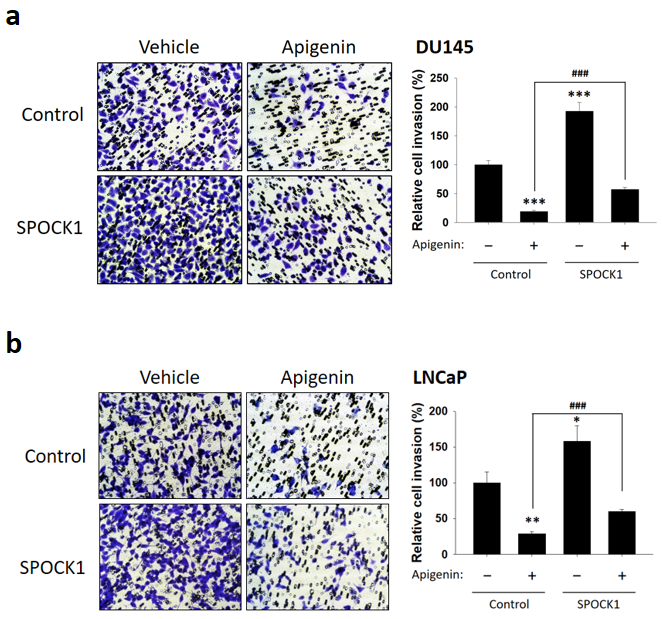
**

**Additional file 1: Figure S3.** SPOCK1 is critical for apigenin (API)-modulated invasiveness of prostate cancer cells. **a, b** Invasive ability of DU145 (**a**) and LNCaP (**b**) cells which were transfected with a vector control or SPOCK1 followed by API or vehicle treatment for an additional 48 h. Left: Representative photomicrographs (200×). Right: Quantitative evaluation of invading cells. Data are presented as the mean ± SD of three independent experiments. * *p*<0.05, ** *p*<0.01, *** *p*<0.001 vs. untreated cells and ^#^ *p*<0.05, ^##^ *p*<0.01, ^###^ *p*<0.001 vs. 40 μM API-treated cells.


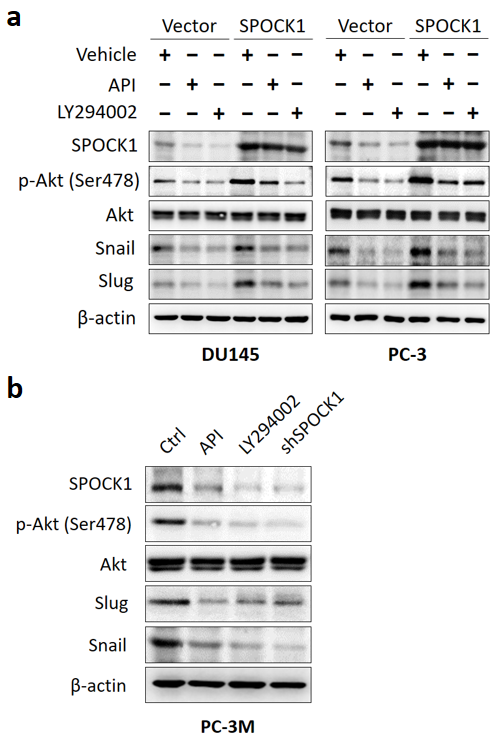


**Additional file 1: Figure S4.** Akt is a downstream regulator of SPOCK1 to regulate Snail family expression in apigenin (API)-treated prostate cancer cells. **a, b** SPOCK1, phosphorylated Akt (Ser473), Akt, Slug, and Snail expression were detected by Western blot analysis in SPOCK1 overexpressing DU145 and PC-3 cells with or without API (40 μM) treatment (**a**) or in PC-3M cells treated with API (40 μM), LY294002 (20 μM) or shSPOCK1 (**b**). β–actin was used as a loading control.

**Additional file 1: Table S1**


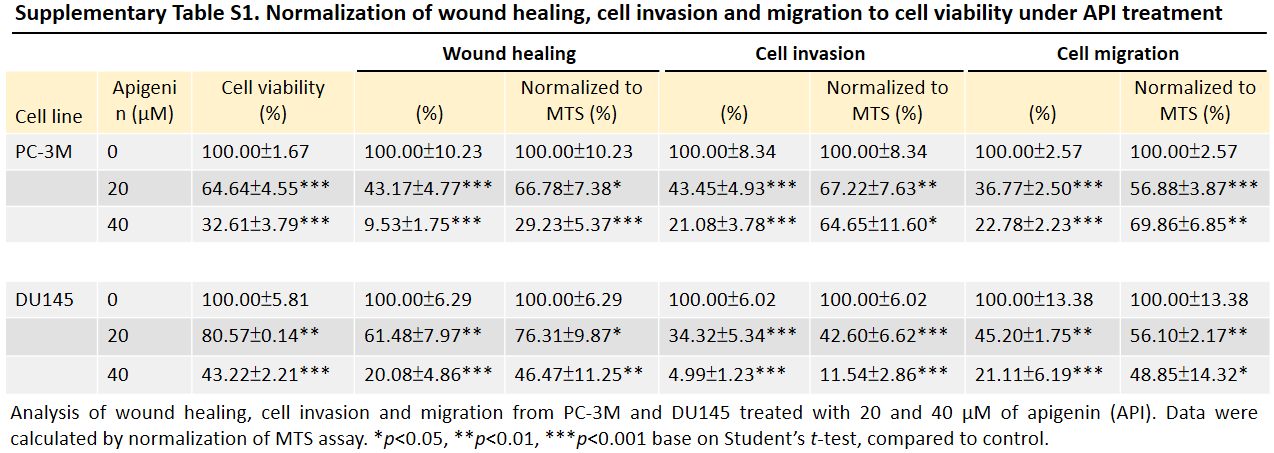

Supplement: Supplementary file 1 — Figure S1. Wound-healing assay of apigenin (API)-treated DU145 prostate cancer cells. Figure S2. Migration and Matrigel invasion assay of DU145 cells using a Transwell system. Figure S3. SPOCK1 is critical for apigenin (API)-modulated invasiveness of prostate cancer cells. Figure S4. Akt is a downstream regulator of SPOCK1 to regulate Snail family expression in apigenin (API)-treated prostate cancer cells. Table S1. Normalization of wound healing, cell invasion and migration to cell viability under API treatment. (DOCX 1043 kb) [file 13046_2019_1247_MOESM1_ESM.docx]
